# The Role of Social Adversity in the Association Between Autistic Traits and Borderline Personality Disorder Symptoms

**DOI:** 10.1002/pmh.70060

**Published:** 2026-01-02

**Authors:** Ellie Roberts, Lucy Scott

**Affiliations:** ^1^ Department of Psychology and Human Development, Institute of Education University College London London UK; ^2^ Department of Mental Health Nursing, Florence Nightingale Faculty of Nursing, Midwifery & Palliative Care King's College London London UK

**Keywords:** adversity, autism, borderline personality disorder, emotionally unstable personality disorder, social support, victimisation

## Abstract

Autism and personality disorders, including borderline personality disorder (BPD), demonstrate high levels of co‐occurrence. Autistic individuals are more likely to experience social adversity and exhibit heightened reactivity to stressors, while social adversity is a well‐established precursor to BPD. The current study investigated the role of social adversity in the association between autistic traits and BPD symptoms, and the moderating role of autistic traits in the association between social adversity and BPD symptoms. Data of 7403 individuals from the 2007 Adult Psychiatric Morbidity Survey in Great Britain were used. Path analysis was conducted to determine whether victimisation and a lack of social support have a role in the association between autistic traits and BPD symptoms. Moderation analysis was applied to assess whether associations of victimisation and a lack of social support with BPD symptoms vary as a function of autistic traits. Analysis was conducted before and after adjustment for sociodemographic covariates. Victimisation and a lack of social support had a role in the relationship between autistic traits and BPD symptoms. Autistic traits moderated the association between victimisation and BPD symptoms, such that the association was greater in individuals with more autistic traits. These observations were robust to adjustment for sociodemographic covariates. The co‐occurrence of BPD in autistic individuals may reflect a double vulnerability, characterised by both heightened exposure to social adversity and increased susceptibility to its effects. However, it is important to note that these findings must be viewed as associational pathways rather than causal relationships.

## 1 Introduction

Autism spectrum disorder (ASD), hereafter ‘autism’, is a neurodevelopmental phenotype characterised by social communication differences and restricted, repetitive behaviours (American Psychiatric Association 2013). Borderline personality disorder (BPD), also known as emotionally unstable personality disorder (EUPD), is a cluster B personality disorder marked by a pervasive instability in affective regulation, interpersonal relationships, self‐image, self‐harm and suicidality, paranoia, feelings of emptiness and a fear of abandonment (American Psychiatric Association 2013). While autism is of neurodevelopmental origin, BPD is often preceded by early trauma, especially of an interpersonal nature, and typically arises in adolescence or early adulthood (American Psychiatric Association 2013). Early traumatic events are strongly linked to BPD aetiology, such that BPD has been suggested to reflect a form of complex post‐traumatic stress disorder, and it has been proposed to be rebranded as a trauma spectrum variant (Lewis and Grenyer 2009). Indeed, up to 90% of individuals with BPD have a history of early adversity (Yen et al. 2002). Types of adversity associated with BPD include physical, sexual and emotional abuse, as well as physical and emotional neglect (Bozzatello et al. 2021).

Approximately half of autistic individuals have been found to meet the diagnostic criteria for at least one personality disorder (Rinaldi et al. 2021). Hofvander et al. (2009) reported that 68% of autistic individuals meet the criteria for one or more personality disorders, 40% for two or more and 18% for three or more. Furthermore, in a sample of autistic women, 45% met the criteria for BPD (Brugha et al. 2020). Autistic individuals with BPD exhibit more frequent suicide attempts, lower global functioning, greater substance abuse and more negative self‐image compared to nonautistic individuals with BPD (Rydén et al. 2008). Associations between specific autistic traits and suicidality in individuals with BPD have been demonstrated. For example, restricted interests, rumination and sensory sensitivity have been found to predict suicidal ideation in those with BPD, while differences in empathy are predictive of suicidal behaviour (Carpita et al. 2025). Approximately 75% of individuals with BPD attempt suicide (Goodman et al. 2017), with an average of three attempts during their lifetime (Soloff et al. 2000), and one in 10 ends their life by suicide (Paris and Zweig‐Frank 2001). Similarly, autistic individuals are 25 times more likely to attempt suicide compared to nonautistic individuals (Conner et al. 2023). The co‐occurrence of autism and BPD is subsequently a significant risk factor for premature mortality.

Similar to individuals with BPD, autistic individuals are more likely to have experienced traumatic events compared to nonautistic individuals (Haruvi‐Lamdan et al. 2018). Notably, these experiences are often interpersonal in nature, such as victimisation and abuse (Beck 2024), as well as stigma, invalidation and rejection (Botha and Frost 2020). The pooled prevalence rate of multiple types of concurrent victimisation in autistic individuals has been reported to be 84% (Trundle et al. 2023). In addition to an increased likelihood of experiencing traumatic events, autistic individuals appear to demonstrate heightened reactivity to stressors. For example, victimised autistic individuals display greater post‐traumatic stress than victimised nonautistic individuals (Paul et al. 2018), and autistic traits are associated with increased rejection sensitivity (Lin et al. 2022). At a physiological level, autistic individuals exhibit heightened and prolonged cortisol reactivity to stressors compared to nonautistic individuals (Spratt et al. 2012). Therefore, being (i) more likely to experience social adversity and (ii) more susceptible to its effects may reflect a type of double vulnerability in autistic individuals regarding the co‐occurrence of trauma‐related disorders such as BPD.

Previous research investigating the association between autism and BPD has focused on co‐occurrence rates and clinical characteristics. Despite established links between autism, social adversity and BPD, no study has assessed the extent to which the association between autism and BPD may involve social adversity. Furthermore, despite evidence suggesting greater stressor reactivity in individuals with autistic traits, to date, no study has examined whether autistic traits may moderate the association between social adversity and BPD symptoms.

The current study sought to address these gaps by using a population‐based cohort to investigate (i) whether the association between autistic traits and BPD symptoms involves social adversity, and (ii) whether the association between social adversity and BPD symptoms is moderated by autistic traits. We hypothesised that (i) the association between autistic traits and BPD symptoms would involve a lack of social support and victimisation, and (ii) the association of a lack of social support and victimisation with BPD symptoms would be moderated by autistic traits, such that the association is greater in individuals with more autistic traits.

## 2 Methods

### 2.1 Participants

Participants were drawn from the 2007 Adult Psychiatric Morbidity Survey in Great Britain (APMS, 2007), conducted between October 2006 and December 2007 by the National Centre for Social Research and the University of Leicester (National Centre for Social Research and the University of Leicester 2024). Using a multistage stratified probability sampling design by region and socioeconomic categories, individuals aged 16 years and above living in a private household were invited to an interview. If there was more than one individual per household, the Kish grid method was employed to randomly select one participant. Interviews were successfully carried out with 7403 individuals, constituting the sample of the current study. Sample weights were used to represent the general population in England by accounting for nonresponse and likelihood of selection. Informed consent was received from all participants. Ethical approval was obtained from the Royal Free Hospital and Medical School of Research Ethics Committee (no. 06/Q0501/71).

### 2.2 Measures

#### 2.2.1 Autistic Traits

The APMS 2007 administered a 20‐item version of the Autism Spectrum Quotient (AQ‐20) (Baron‐Cohen et al. 2001) to minimise nonresponse (Brugha et al. 2009). Each item assessed an autistic trait and was scored dichotomously as 0 (no) or 1 (yes), resulting in a total score ranging from 0 to 20, with higher scores reflecting the presence of more autistic traits. This 20‐item version of the AQ has previously been used to investigate autistic traits (Martinez et al. 2021).

#### 2.2.2 BPD Symptoms

Symptoms of BPD were assessed using the SCID‐II PQ‐BPD questionnaire, a screening tool used in outpatient settings that demonstrates good psychometric properties (Chanen et al. 2008). The questionnaire contains 15 items, each scored 0 (no) or 1 (yes) to indicate the presence of a symptom. Total scores therefore range from 0 to 15, with higher scores representing a greater number of symptoms.

#### 2.2.3 Social Adversity

Multiple social experiences were applied in an exploratory factor analysis, which yielded two factors representing victimisation and a lack of social support. The victimisation factor included lifetime measures of bullying, physical abuse at home and sexual abuse, each with responses of no (0) or yes (1). Measures of social support comprised the statements: ‘family and friends do things to make me happy’, ‘family and friends make me feel loved’, ‘family and friends can be relied on no matter what happens’, ‘family and friends would see that I am taken care of’, ‘family and friends accept me just the way I am’, ‘family and friends make me feel an important part of their lives’ and ‘family and friends give me support and encouragement’. Each statement was scored not true (0), partly true (1) or certainly true (2). The responses were reverse coded in the current study and constituted the lack of social support factor.

#### 2.2.4 Covariates

Potential covariates of age (continuous), sex (female and male), ethnicity (white and non‐white) and socioeconomic deprivation were controlled for. Socioeconomic deprivation was measured using a five‐category Index of Multiple Deprivation (IMD), with 1 representing the least deprived areas and 5 the most deprived areas.

### 2.3 Analytic Method

Analysis was conducted in STATA 18. Descriptive statistics summarised the characteristics of the sample. Exploratory factor analysis using promax rotation was used to assess dimensions within the social experience measures. Path analysis was conducted to investigate associational pathways between the identified factors (victimisation and lack of social support), autistic traits and BPD symptoms. Moderation analysis was applied to evaluate the impact of autistic traits on the association between the social adversity factors and BPD symptoms. All regression analysis was weighted and carried out before and after adjustment for covariates. Full information maximum likelihood was applied to handle missing data. Variables were not standardised or centred prior to model estimations, as scales did not largely differ. Sensitivity analysis involving an antisocial personality disorder (ASPD) measure (the only other personality disorder measured by APMS), SCID‐II PQ‐ASPD, was conducted to investigate whether findings could be generalised to broader personality pathology (combined BPD and ASPD scores).

## 3 Results

### 3.1 Descriptive Statistics

Descriptive statistics can be seen in Table 1. The average age of the sample was 51.12 years (SD = 18.59). The sample was relatively equally divided by sex (56.81% female) but was predominantly of white ethnicity (92.57%). The most common IMD was 8.35–13.72, representing the second to least deprived areas. The average AQ‐20 score was 5.95 (SD = 2.58) and the average SCID‐II PQ‐BPD score was 1.81 (SD = 2.66).

**TABLE 1:** Descriptive statistics of the sample, unweighted (*N* = 7403).

Variable	*N* (%) or *N*, Mean (M) (SD)
Age	*N* = 7403, M = 51.12 (SD = 18.59)
*Sex*	
Female	*N* = 4206 (56.81%)
Male	*N* = 3197 (43.19%)
*Ethnicity*	
White	*N* = 6807 (92.57%)
Non‐white	*N* = 546 (7.43%)
*Index of Multiple Deprivation (IMD)*	
0.59–8.35 (least deprived)	*N* = 1418 (19.15%)
8.35–13.72	*N* = 1639 (22.14%)
13.72–21.16	*N* = 1467 (19.82%)
21.16–34.21	*N* = 1387 (18.74%)
34.21–86.36 (most deprived)	*N* = 1492 (20.15%)
Autistic traits (AQ‐20)	*N* = 7377, M = 5.95 (SD = 2.58)
BPD traits (SCID‐II PQ‐BPD)	*N* = 6213, M = 1.81 (SD = 2.66)
ASPD traits (SCID‐II PQ‐ASPD)	*N* = 6553, M = 0.96 (SD = 1.56)
**Victimisation**	
*Bullied*	
Yes	*N* = 1392 (18.92%)
No	*N* = 5965 (81.08%)
*Physical abuse*	
Yes	*N* = 695 (9.45%)
No	*N* = 6662 (90.55%)
*Sexual abuse*	
Yes	*N* = 386 (5.25%)
No	*N* = 6971 (94.75%)
**Lack of social support**	
*Not accepted by others*	
Not true	*N* = 6831 (92.95%)
Partly true	*N* = 464 (6.31%)
Certainly true	*N* = 54 (0.73%)
*Do not feel loved*	
Not true	*N* = 6531 (88.88%)
Partly true	*N* = 712 (9.69%)
Certainly true	*N* = 105 (1.43%)
*Others do not do things to make me happy*	
Not true	*N* = 6188 (84.18%)
Partly true	*N* = 1032 (14.04%)
Certainly true	*N* = 131 (1.78%)
*Cannot rely on others*	
Not true	*N* = 6626 (90.17%)
Partly true	*N* = 624 (8.49%)
Certainly true	*N* = 98 (1.33%)
*Not taken care of by others*	
Not true	*N* = 6698 (91.25%)
Partly true	*N* = 556 (7.57%)
Certainly true	*N* = 86 (1.17%)
*Do not feel important to others*	
Not true	*N* = 6337 (86.29%)
Partly true	*N* = 880 (11.98%)
Certainly true	*N* = 127 (1.73%)
*Do not receive support or encouragement*	
Not true	*N* = 6501 (88.46%)
Partly true	*N* = 746 (10.15%)
Certainly true	*N* = 102 (1.39%)

### 3.2 Factor Analysis

Exploratory factor analysis using promax rotation was applied to uncover dimensions within the social experience measures. Horn's parallel analysis was conducted to determine the most suitable number of factors (see Table S1). The loadings resulted most fittingly in a two‐factor solution representing experiences of lacking social support and being victimised. Table 2 displays factor loadings > 0.32 highlighted. The two factors were weakly correlated (*r* = 0.233; *p* < 0.001). Values for Cronbach's alpha were 0.90 for the lack of social support factor and 0.74 for the victimisation factor. Regarding model fit indices for the lack of social support factor, the RMSEA (0.06), CFI (0.99), TLI (0.98) and SRMR (0.02) indicated excellent fit. Due to the victimisation factor only including three items, the model was just‐identified, as expected. Subsequently, fit indices are not informative.

**TABLE 2:** Factor loadings for lack of support and victimisation after promax rotation.

Variables	Factor loadings	
	Lack of social support	Victimisation
Not accepted by others	**0.6474**	0.0908
Do not feel loved	**0.7598**	−0.0673
Others do not do things to make me happy	**0.6388**	−0.0558
Cannot rely on others	**0.7573**	0.0179
Not taken care of by others	**0.7462**	0.0219
Do not feel important to others	**0.7753**	0.0152
Do not receive support or encouragement	**0.8086**	−0.0058
Bullied	−0.0117	**0.4219**
Physical abuse	0.0101	**0.5319**
Sexual abuse	−0.0027	**0.4581**

*Note:* The numbers in bold represent those with factor loadings above 0.32.

### 3.3 Path Analysis

**FIGURE 1:**
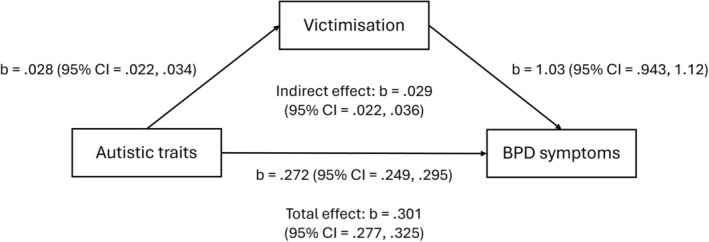
Diagram visualising associations between autistic traits, victimisation and BPD symptoms observed in path analysis.

**FIGURE 2:**
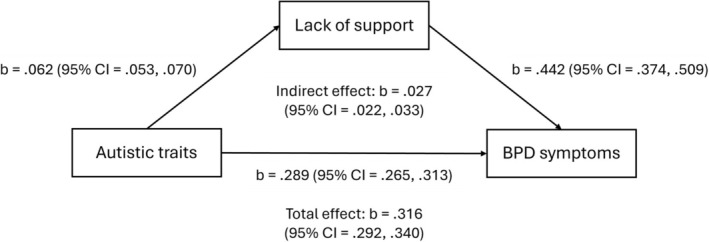
Diagram visualising associations between autistic traits, lack of support and BPD symptoms observed in path analysis.

Results from path analysis investigating the role of victimisation and lack of social support in the association between autistic traits and BPD symptoms can be seen in Table 3. There was a significant associational pathway involving victimisation (adjusted model: b = 0.029; 95% CI = 0.022, 0.036; *p* < 0.001) and lack of social support (adjusted model: b = 0.027; 95% CI = 0.022, 0.033; *p* < 0.001). These associations are visualised in Figures 1 and 2. The same findings were observed in sensitivity analysis with broader personality pathology (see Table S2).

**TABLE 3:** Path analysis testing the role of victimisation and lack of social support in the association between autistic traits and BPD symptoms.

Autistic traits (AT) ➔ victimisation ➔ BPD symptoms			
*Unadjusted model*			
	**b**	**95% CI**	***p*‐value**
AT ➔ BPD	0.228	0.205, 0.252	< 0.001
AT ➔ victim	0.028	0.022, 0.034	< 0.001
Victim ➔ BPD	1.30	1.21, 1.38	< 0.001
Indirect effect	0.036	0.028, 0.045	< 0.001
Total effect	0.265	0.240, 0.290	< 0.001
*Adjusted model*			
	**b**	**95% CI**	***p*‐value**
AT ➔ BPD	0.272	0.249, 0.295	< 0.001
AT ➔ victim	0.028	0.022, 0.034	< 0.001
Victim ➔ BPD	1.03	0.943, 1.12	< 0.001
Indirect effect	0.029	0.022, 0.036	< 0.001
Total effect	0.301	0.277, 0.325	< 0.001

Autistic traits (AT) ➔ lack of social support (LS) ➔ BPD symptoms			
*Unadjusted model*			
	**b**	**95% CI**	***p*‐value**
AT ➔ BPD	0.237	0.212, 0.262	< 0.001
AT ➔ LS	0.062	0.053, 0.070	< 0.001
LS ➔ BPD	0.467	0.395, 0.538	< 0.001
Indirect effect	0.029	0.023, 0.035	< 0.001
Total effect	0.266	0.241, 0.291	< 0.001
*Adjusted model*			
	**b**	**95% CI**	***p*‐value**
AT ➔ BPD	0.289	0.265, 0.313	< 0.001
AT ➔ LS	0.062	0.053, 0.070	< 0.001
LS ➔ BPD	0.442	0.374, 0.509	< 0.001
Indirect effect	0.027	0.022, 0.033	< 0.001
Total effect	0.316	0.292, 0.340	< 0.001

### 3.4 Moderation Analysis

**FIGURE 3:**
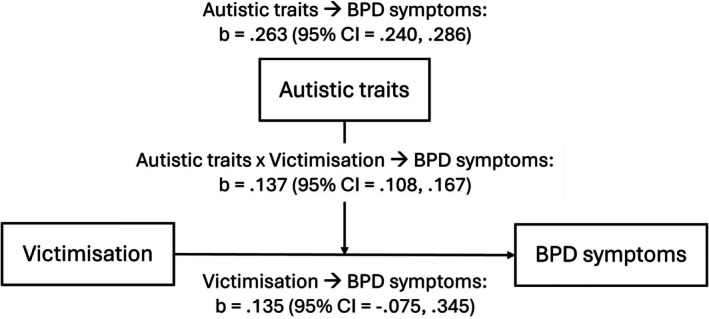
Diagram visualising the moderating role of autistic traits in the association between victimisation and BPD symptoms.

Results from moderation analysis are shown in Table 4. Autistic traits moderated the association between victimisation and BPD symptoms (adjusted model: b = 0.137; 95% CI = 0.108, 0.167; *p* < 0.001), such that the association was greater in those with more autistic traits. This moderation is visualised in Figure 3. No moderation in the pathway from a lack of social support was found. The same findings were observed in sensitivity analysis with broader personality pathology (see Table S3).

**TABLE 4:** Moderation analysis testing interactions between autistic traits and social adversity in associations with BPD symptoms.

Victimisation ➔ BPD symptoms			
*Unadjusted model*			
	**b**	**95% CI**	***p*‐value**
Victim ➔ BPD	0.252	0.033, 0.471	0.024
AT ➔ BPD	0.219	0.196, 0.243	< 0.001
AT × victim ➔ BPD	0.159	0.129, 0.190	< 0.001
*Adjusted model*			
	**b**	**95% CI**	***p*‐value**
Victim ➔ BPD	0.135	−0.075, 0.345	0.208
AT ➔ BPD	0.263	0.240, 0.286	< 0.001
AT × victim ➔ BPD	0.137	0.108, 0.167	< 0.001

Lack of social support (LS) ➔ BPD symptoms			
*Unadjusted model*			
	**b**	**95% CI**	***p*‐value**
LS ➔ BPD	0.215	0.020, 0.410	0.031
AT ➔ BPD	0.237	0.212, 0.262	< 0.001
AT × LS ➔ BPD	0.036	0.010, 0.063	0.007
*Adjusted model*			
	**b**	**95% CI**	***p*‐value**
LS ➔ BPD	0.362	0.179, 0.545	< 0.001
AT ➔ BPD	0.289	0.265, 0.313	< 0.001
AT × LS ➔ BPD	0.012	−0.013, 0.036	0.354

## 4 Discussion

The current study sought to investigate whether the association between autistic traits and BPD symptoms is related to social adversity, and whether autistic traits moderate the association between social adversity and BPD symptoms. Results indicated that both victimisation and a lack of social support were involved in the association between autistic traits and BPD symptoms, aligning with our initial hypothesis. Due to the cross‐sectional nature of the path analysis, findings should be interpreted as associational pathways, not causal processes (Weems 2025). In addition, autistic traits moderated the association between victimisation and BPD symptoms, such that the pathway from victimisation was greater in individuals with more autistic traits, also aligning with our hypothesis. This could support the proposition that individuals with increased autistic traits are more vulnerable to the effects of social adversity such as victimisation. Nevertheless, we did not observe the same moderating effect by autistic traits in the pathway from a lack of social support, contrary to our hypothesis. All observations were robust to adjustment for sociodemographic covariates. These findings may evidence the idea of a double vulnerability regarding autistic traits, whereby individuals with higher autistic traits experience increased social adversity and are more susceptible to its effects, both contributing to heightened BPD symptoms.

While previous research has established associations between autism, social adversity and BPD, this study is the first to demonstrate a role for social adversity in the association between autistic traits and BPD symptoms. Differences in social behaviour and communication have been hypothesised to drive increases in victimisation of autistic individuals by nonautistic individuals, including peers and caregivers (Beck 2024). Autistic individuals report that discrimination and social rejection due to being misunderstood by nonautistic individuals are significant contributors to their experiences of emotional dysregulation and low self‐worth (Beck et al. 2024). Pathways from adversity to BPD have been proposed to involve dysregulation of the hypothalamic–pituitary–adrenal (HPA) axis, changes in grey matter volume such as hippocampal reduction, epigenetic modifications affecting the expression of neurotransmitters and stress hormones and increased amygdala activation (Bozzatello et al. 2021). The development of maladaptive cognitive schemas following interpersonal trauma may also play a role, as individuals with BPD display schemas surrounding mistrust and defectiveness (Bach and Farrell 2018). Considering the current findings, autistic individuals may experience repetitive social adversity such as discrimination, victimisation and rejection, which contribute to the development of BPD symptoms via alterations in neural structure and function as well as cognitive frameworks.

The observation that the association between victimisation and BPD symptoms is greater in those with increased autistic traits aligns with previous research indicating that autistic individuals experience greater sensitivity to social stressors than nonautistic individuals. This has been shown in response to peer victimisation (Paul et al. 2018) and rejection‐induced social pain (Lin et al. 2022; Trimmer et al. 2017). This sensitivity may arise from repeated experiences of rejection (Beck 2024), consistent with the observations of Chen et al. (2018) that victimisation increases cortisol reactivity to social stress. Rejection sensitivity is also frequently observed in individuals with BPD (Foxhall et al. 2019).

While autistic traits moderated the association between victimisation and BPD symptoms, a moderating effect for lack of social support was not observed. This absence of a moderation effect may reflect differences in the nature of these types of social adversity. Victimisation (comprising bullying, physical abuse and sexual abuse in the current study) could represent an especially stressful and threatening experience with greater acute physiological arousal. Considering that autistic individuals demonstrate higher levels of perceived stress (Ilen et al. 2024) and increased cortisol reactivity to stressful situations (Spratt et al. 2012), heightened sensitivity to stress as well as stress reactivity may account for the variability in the impact of victimisation on BPD symptoms across the levels of autistic traits, making moderation more detectable than for social support. A lack of social support is likely more stable than victimisation, as well as potentially having less variation in severity and impact. This factor may have a role that is more mechanistic rather than conditional on autistic traits. This pattern may therefore align with the diathesis–stress model, where autistic traits function as a vulnerability factor, while victimisation acts as an environmental stressor, which amplifies this vulnerability.

Cohen and Wills (1985) identified that buffering effects, where support protects individuals from the negative effects of stress, occurred only when support measures were directly relevant to the stressor, while global measures, such as low social support, exerted a largely uniform effect across individuals, regardless of independent characteristics. Consistent with this, our measure of social support provides a broad indicator of one's interpersonal environment, while victimisation reflects an acute and socially complex form of adversity. Therefore, the use of social support as a global measure may explain the lack of interaction with autistic traits.

It is important to note that personality disorders have high rates of co‐occurrence, implying that they could be combined under a general factor of personality pathology (Sharp et al. 2015). It has been proposed that personality pathology is best understood as a general factor representing overall severity, in addition to specific factors differentiating types of dysfunctions (Sharp et al. 2015). Recent recommendations have been made to shift the approach to personality pathology from categorical (i.e., separate diagnoses) to a dimensional, severity‐based conceptualisation (Tyrer et al. 2025). In the current study, we were only able to investigate the presence of BPD symptoms, which, although a more dimensional approach than binary diagnosis, does not capture severity. The sensitivity analysis incorporating scores from an ASPD measure demonstrated that results held with this more general measure of personality pathology. However, because both BPD and ASPD are cluster B personality disorders, these associations may imply that our observed effects are only specific to cluster B personality pathology (not general personality pathology). Future studies could therefore seek to confirm this with a more diverse and comprehensive measure of general personality pathology.

The current study has important implications for public health. Addressing experiences of social adversity may be an effective intervention in the prevention and treatment of BPD symptoms in individuals with autistic traits. Importantly, social adversity can include both victimisation and a lack of social support. Autistic peer support has been reported to foster belonging and acceptance (Huang et al. 2024). Ideally, such interventions are implemented early to prevent the cumulative effects of repeated social adversity. Autistic students have demonstrated positive perceptions of school‐based autistic peer support, including finding likeminded peers, inclusivity, acceptance and understanding (Crompton et al. 2023). These interventions may provide unique environments in which autistic individuals can feel supported and accepted. Regarding victimisation, especially bullying, interventions, which prioritise educating nonautistic individuals about autism, may be beneficial. Such interventions have been evidenced to increase knowledge and acceptance and decrease stigma towards autistic individuals (Gillespie‐Lynch et al. 2015; Gillespie‐Lynch et al. 2022). Mitigating experiences of abuse and violence may involve education about rights, the definition of consent and violence and how to access support services (Gibbs et al. 2023).

While educating nonautistic peers about bullying and social differences is essential, the responsibility of reducing victimisation should not fall solely on autistic individuals. Complementing these preventative and systemic efforts, targeted screening for trauma and social adversity in autistic individuals may play a critical role in identifying autistic individuals at heightened risk for developing BPD symptoms, therefore informing tailored interventions. While initially developed for individuals diagnosed with BPD, mentalisation‐based therapy (MBT), which focuses on enhancing the capacity to understand one's own and others' mental states (Vogt and Norman 2019), offers a clinically grounded intervention for these at‐risk individuals. Indeed, Krämer et al. (2021) identified MBT as a promising intervention that improves mentalising abilities in autistic individuals. Therefore, by improving mentalisation, MBT may address the effects of social adversity, improving emotion regulation and relational functioning. Early identification through targeted screening allows for timely, adapted MBT interventions, potentially mitigating the emergence of BPD symptoms in autistic populations. This may help to support autistic individuals in navigating social challenges without placing the burden of systemic change solely on them.

However, one limitation of the current study is that the measures of social adversity, in particular victimisation, were interpreted as binary variables. Victimisation likely encompasses a wide range of experiences, which vary in type, severity, frequency and chronicity, and it is impossible to capture this heterogeneity with dichotomous coding. Although dichotomising variables can attenuate effect sizes and produce conservative associations (MacCallum et al. 2002), a significant association was observed between victimisation and BPD symptoms, and this association is moderated by autistic traits. This suggests the underlying relationships are robust and may even be stronger when assessed using dimensional measures. Future research should seek to employ more dimensional measures of social adversity, which would allow for a more nuanced understanding of the role of this variable in the association between autistic traits and BPD symptoms.

Further, it is important to consider that the wide age range of the sample introduces possible developmental influences on the assessment of BPD symptoms. Although the SCID‐II PQ‐BPD assesses lifetime symptoms, symptoms of BPD typically peak in early adulthood and attenuate with age, mirroring aspects of typical development (Winsper 2021). Considering this, it is possible that older participants may underreport past symptoms, leaving the current measure to reflect a phenotype shaped by attenuated symptomology or age‐related change. This may influence both the observed prevalence of BPD symptoms and the magnitude of its association with social adversity and autistic traits. Longitudinal studies employing age‐stratified analyses will be useful in clarifying the potential variation in such associations across the lifespan.

Given the cross‐sectional design of the current study, the directionality of associations remains uncertain and causal interpretations are not possible. However, autism is a neurodevelopmental phenotype with traits observable from early childhood, whereas BPD symptoms typically emerge in adolescence or early adulthood (American Psychiatric Association 2013). Moreover, autistic individuals often experience social adversity from an early age, such as during primary school (Junttila et al. 2024), and there is a greater body of evidence reporting that BPD symptoms are preceded by adversity, rather than the reverse. Nevertheless, it must be emphasised that findings from path analysis as employed in the current study cannot be interpreted as links to causation, but rather associational pathways. It should also be noted that the effects we found were very small, which limits inferences of a double vulnerability. Furthermore, the potential for reciprocal, bidirectional associations must be considered. For example, BPD may contribute to further susceptibility to social adversity among those with autistic traits, a phenomenon which has previously been observed (Bagge et al. 2004). Future research incorporating cross‐lagged panel models may capture the more complex associations between these variables. Another important consideration is that autism and BPD share many similarities and overlapping features such as emotional dysregulation and interpersonal difficulties (Dell'Osso et al. 2023), raising the possibility that BPD symptoms may partly reflect an expression of autistic traits. Further research should seek to replicate the current findings using a longitudinal design that accounts for BPD symptoms before the occurrence of social adversity. While autistic peer support and MBT for autistic populations represent interventions in ongoing development, future research could investigate whether such interventions are effective in the prevention and mitigation of BPD symptoms.

## 5 Conclusion

Experiences of victimisation and a lack of social support are involved in the association between autistic traits and BPD symptoms. Additionally, autistic traits moderate the association between victimisation and BPD symptoms, with stronger associations observed in individuals with more autistic traits. Together, these findings may contribute to evidencing the idea of a double vulnerability regarding the co‐occurrence of BPD symptoms, whereby individuals with increased autistic traits are both more likely to experience social adversity and more susceptible to its effects.

## Funding

This work was supported by the Medical Research Council (MRC) (grant reference: MR/W006774/1).

## Ethics Statement

Ethical approval was obtained from the Royal Free Hospital and Medical School of Research Ethics Committee (no. 06/Q0501/71). Informed consent was received from all participants.

## Conflicts of Interest

The authors declare no conflicts of interest.

## Supporting information


**Table S1:** Results from Horn's parallel analysis for principal components.
**Table S2:** Path analysis testing the role of victimisation and lack of social support in the association between autistic traits and personality pathology.
**Table S3:** Moderation analysis testing interactions between autistic traits and social adversity in associations with personality pathology.

## Data Availability

Data are available in a public, open access repository. UK Data Service. National Centre for Social Research, University of Leicester. (2025). *Adult Psychiatric Morbidity Survey, 2007*. [data collection]. *4th Edition*. UK Data Service. SN: 6379, DOI: https://doi.org/10.5255/UKDA‐SN‐6379‐2.
