# Supplementary material for: The Role of Social Adversity in the Association Between Autistic Traits and Borderline Personality Disorder Symptoms
Source: Personal Ment Health. 2026 Jan 2;20(1):e70060. doi: 10.1002/pmh.70060 (PMC12758095; doi:10.1002/pmh.70060)
Supplement: Supplementary file 1 — Table S1: Results from Horn's parallel analysis for principal components. Table S2: Path analysis testing the role of victimisation and lack of social support in the association between autistic traits and personality pathology. Table S3: Moderation analysis testing interactions between autistic traits and social adversity in associations with personality pathology. [file PMH-20-0-s001.docx]

**Supplementary Material**

**Supplementary Table 1.** Results from Horn’s parallel analysis for principal components

| **Component/factor** | **Eigenvalue** |
| --- | --- |
| 1 | 4.208 |
| 2 | 1.381 |
| 3 | 0.784 |
| 4 | 0.734 |
| 5 | 0.671 |

**Supplementary Table 2.** Path analysis testing the role of victimisation and lack of social support in the association between autistic traits and personality pathology

| **Autistic Traits (AT) 🡪 Victimisation 🡪 Personality Pathology (PP)** | | | |
| --- | --- | --- | --- |
| *Unadjusted model* | | | |
|  | **b** | **95% CI** | **p-value** |
| AT 🡪 PP | .279 | .017 | <.001 |
| AT 🡪 Victim | .028 | .003 | <.001 |
| Victim 🡪 PP | 2.01 | .064 | <.001 |
| Indirect effect | .057 | .007 | <.001 |
| Total effect | .336 | .018 | <.001 |
| *Adjusted model* | | | |
|  | **b** | **95% CI** | **p-value** |
| AT 🡪 PP | .316 | .017 | <.001 |
| AT 🡪 Victim | .028 | .003 | <.001 |
| Victim 🡪 PP | 1.69 | .063 | <.001 |
| Indirect effect | .047 | .006 | <.001 |
| Total effect | .364 | .017 | <.001 |
| **Autistic Traits (AT) 🡪 Lack of Social Support (LS) 🡪 Personality Pathology (PP)** | | | |
| *Unadjusted model* | | | |
|  | **b** | **95% CI** | **p-value** |
| AT 🡪 PP | .291 | .018 | <.001 |
| AT 🡪 LS | .062 | .004 | <.001 |
| LS 🡪 PP | .714 | .053 | <.001 |
| Indirect effect | .044 | .004 | <.001 |
| Total effect | .335 | .018 | <.001 |
| *Adjusted model* | | | |
|  | **b** | **95% CI** | **p-value** |
| AT 🡪 PP | .346 | .017 | <.001 |
| AT 🡪 LS | .062 | .004 | <.001 |
| LS 🡪 PP | .650 | .049 | <.001 |
| Indirect effect | .040 | .004 | <.001 |
| Total effect | .386 | .017 | <.001 |

**Supplementary Table 3.** Moderation analysis testing interactions between autistic traits and social adversity in associations with personality pathology

| **Victimisation 🡪 Personality Pathology (PP)** | | | |
| --- | --- | --- | --- |
| *Unadjusted model* | | | |
|  | **B** | **95% CI** | **p-value** |
| Victim 🡪 PP | .776 | .161 | <.001 |
| AT 🡪 PP | .270 | .017 | <.001 |
| AT x Victim 🡪 PP | .189 | .023 | <.001 |
| *Adjusted model* | | | |
|  | **b** | **95% CI** | **p-value** |
| Victim 🡪 PP | .642 | .152 | <.001 |
| AT 🡪 PP | .308 | .017 | <.001 |
| AT x Victim 🡪 PP | .161 | .021 | <.001 |
| **Lack of Social Support (LS) 🡪 Personality Pathology (PP)** | | | |
| *Unadjusted model* | | | |
|  | **b** | **95% CI** | **p-value** |
| LS 🡪 PP | .515 | .146 | <.001 |
| AT 🡪 PP | .291 | .018 | <.001 |
| AT x LS 🡪 PP | .029 | .019 | 0.142 |
| *Adjusted model* | | | |
|  | **b** | **95% CI** | **p-value** |
| LS 🡪 PP | .630 | .135 | <.001 |
| AT 🡪 PP | .346 | .017 | <.001 |
| AT x LS 🡪 PP | .003 | .018 | 0.874 |
